# Laboratory assessment of trilostane treatment in dogs with pituitary‐dependent hyperadrenocorticism

**DOI:** 10.1111/jvim.15830

**Published:** 2020-06-13

**Authors:** Carolina Arenas Bermejo, Dolores Pérez Alenza, Paula García San José, Lidia Llauet, Laura Pérez‐López, Carlos Melián, Edward C. Feldman

**Affiliations:** ^1^ Internal Medicine Service, Anicura Hospital Veterinario Valencia Sur Valencia Spain; ^2^ Department of Animal Medicine and Surgery, Veterinary Faculty Complutense University of Madrid Madrid Spain; ^3^ Internal Medicine Service, Hospital Veterinari Catalunya Odena Spain; ^4^ Department of Animal Pathology, University of Las Palmas de Gran Canaria Las Palmas de Gran Canaria Spain; ^5^ Department of Medicine and Epidemiology, School of Veterinary Medicine University of California Davis California USA

**Keywords:** adrenal, canine, Cushing syndrome, treatment

## Abstract

**Background:**

Results of ACTH stimulation test (ACTHst), pre‐ and post‐trilostane serum cortisol concentrations (SCCs), urine concentration (urine‐specific gravity [USG]), and urine cortisol : creatinine ratios (UCCRs) are common variables used to monitor trilostane treatment of dogs with pituitary‐dependent hyperadrenocorticism (PDH). However, none has consistently discriminated dogs receiving an adequate dose (A) from those overdosed (O) or underdosed (U).

**Objectives:**

To assess and compare recommended monitoring variables, including serial SCCs in a cohort of dogs with PDH treated with trilostane.

**Animals:**

Privately owned dogs with PDH (n = 22) and 3 healthy dogs (controls).

**Methods:**

Prospective, multicenter, 2‐day study. On day “a” (randomized): ACTHst was completed. Day “b” (>2 to <7 days later): SCCs were assessed −0.5 hours, immediately before, and 1, 2, 2.5, 3, 3.5, 4, 6, 8, and 12 hours after trilostane administration. On the first study day, urine collected at home was assessed for USG, UCCR and owner opinions regarding PDH were categorized as: A (clinical signs resolved), U (remains symptomatic), or ill (possible O).

**Results:**

At 27 pairs of evaluations, 7 dogs were categorized as A, 19 U, and 1 possible O (excluded from the study). There was overlap in SCC results from the A and U dogs at every time point. Results of USG, UCCR, and ACTHst did not discriminate A from U dogs. Trilostane suppresses SCC within 1 hour of administration and its duration of action in most PDH dogs is <8 hours.

**Conclusions and Clinical Importance:**

No single variable or group of variables reliably discriminated A dogs from U dogs during trilostane treatment for PDH.

**Abbreviations**
A: adequate dose
ACTHst: ACTH stimulation test
eACTH: endogenous ACTH
G: good dose
HAC: hyperadrenocorticism
LDDST: low dose dexamethasone suppression test
O: overdosed
PD: polydipsia
PDH: pituitary‐dependent hyperadrenocorticism
PP: polyphagic
PU: polyuria
SCCs: serum cortisol concentrations
U: underdosed
UCCRs: urine cortisol : creatinine ratios
USG: urine‐specific gravity

## 1 INTRODUCTION

Between 1970 and 2000, the most common medical treatment for dogs with pituitary‐dependent hyperadrenocorticism (PDH) was mitotane (o,p'‐DDD), a cytotoxic drug that targets adrenocortical cells.^1^, ^2^, ^3^, ^4^ Since 2000, the most common medical treatment for dogs with PDH has been trilostane, a competitive inhibitor of the 3β‐hydroxysteroid dehydrogenase‐isomerase enzyme system.^5^, ^6^, ^7^, ^8^, ^9^, ^10^, ^11^, ^12^

Several different mitotane protocols were suggested for dogs with PDH.^3^, ^4^ Regardless of protocol, authors consistently considered ACTH stimulation test (ACTHst) results to be a reliable objective indicator of mitotane overdose, underdose, or adequate dosage, regardless of when the test was begun relative to time of previous mitotane administration.^1^, ^2^, ^3^, ^4^ Perhaps because results of the ACTHst were considered so reliable in monitoring mitotane treatment, the test began being used for the same purpose in trilostane‐treated dogs in initial reports ^8^, ^9^, ^10^, ^11^ and in the manufacturer's insert.^12^ Subsequent studies, however, indicated that ACTHst results were influenced by when the test was started relative to the most recent trilostane administration.^13^, ^14^, ^15^, ^16^ The drug manufacturer recommends beginning the ACTHst 4 to 6 hours post‐trilostane administration,^12^ but results obtained with tests started 4 hours after trilostane administration are likely to be different from those obtained when the test is begun 6 hours after, regardless of dose.^15^, ^17^ Although no study has validated the 4 to 6 hour recommendation, it has been reported that results of ACTHst initiated 3 hours after trilostane administration were significantly different from tests started 9 hours after, and those started 2 hours after were significantly different from those started 4 hours after.^14^, ^15^ Completely different decisions regarding maintaining, increasing, or decreasing trilostane dose or frequency could be the result of test timing. Adding to the confusion, suggested ACTHst starting times in published studies have varied from as early as 2 hours to as late as 12 or 24 hours after trilostane administration.^13^, ^14^, ^15^, ^16^, ^17^, ^18^ Regardless of timing issues, and perhaps more important, concerns persist that ACTHst results do not reliably indicate which dogs are overdosed, underdosed, or dosed adequately.^13^, ^14^, ^16^, ^19^, ^20^

With so many issues raised regarding the ACTHst, it is not surprising that alternative monitoring variables have been investigated, including endogenous ACTH (eACTH), cortisol : eACTH ratios, urine cortisol : creatinine ratios (UCCRs), and circulating baseline serum cortisol concentrations (SCCs) before or after trilostane administration or both.^17^, ^20^, ^21^, ^22^, ^23^, ^24^, ^25^, ^26^ None of these variables has provided consistently sensitive and specific indications for discriminating dogs adequately dosed from those receiving too little or too much medication. With failure to identify a gold standard for objective monitoring of trilostane treatment, it is also fair to suggest that veterinary clinicians using trilostane to treat PDH either do not have confidence in any proposed monitoring variable or have placed their confidence in a variable that may not reliably provide the information they seek. Our aim was to assess several previously reported and promoted objective variables as well as serial circulating SCCs in a group of dogs with confirmed PDH given trilostane, and categorized subjectively by their owners as receiving an “adequate dose” (A) or being “underdosed” (U). Our hypothesis was that only urinespecific gravity (USG) from trilostane‐treated dogs would consistently discriminate those dosed adequately from underdosed dogs.

## 2 MATERIALS AND METHODS

### 2.1 Dogs

Healthy control dogs and dogs with confirmed PDH were prospectively recruited from 3 veterinary hospitals in Spain (Veterinary Teaching Hospital, University Complutense, Madrid; Veterinary Hospital, University Las Palmas de Gran Canaria, Grand Canary Island; and Aúna Especialidades Veterinarias, Valencia) after obtaining informed consent from their owners. Health of the control dogs based on history, physical examination findings, and results of CBC, routine serum biochemistry and urinalysis that were within the reference range. In each PDH dog, hyperadrenocorticism (HAC) initially would be suspected from a review of historical information and physical examination findings. All PDH dogs must have had polyuria (PU) and polydipsia (PD) as owner concerns and either have been described as having excellent appetite or being polyphagic (PP). Each must have had at least 4 of the following 6 clinicopathologic findings: increased serum alkaline phosphatase activity, alanine aminotransferase activity, and serum cholesterol concentration; low BUN concentration or BUN concentration near the lower reference limit (low normal result); USG < 1.020; and no microbial growth on bacteriologic culture of urine. No dog had a BUN concentration above the reference interval. No dog had evidence of any serious illness other than PDH, including diabetes mellitus, chronic kidney disease, or neurologic signs consistent with a large pituitary mass. Results of a low dose dexamethasone suppression test (LDDST) or ACTHst must have been consistent with the diagnosis of HAC in each dog; not all dogs underwent both tests. Diagnosis of PDH was made if a dog had at least 2 of the following: a LDDST result indicative of PDH, ultrasonographic evidence of 2 relatively equal‐sized adrenal glands, or plasma concentrations of eACTH > 45 pg/mL (reference range, 20‐80 pg/mL).^27^, ^28^, ^29^, ^30^, ^31^, ^32^

### 2.2 Trilostane treatment

The initial trilostane dosage for each PDH dog enrolled was 0.5 to 1.1 mg/kg PO q12h. Each dog must have been treated for a minimum of 30 days and their doses adjusted at previous visits, if such visits were made, in an attempt to achieve clinical control. Commercially available trilostane (Vetoryl, Dechra, Shrewsbury, UK) was used and, if needed, capsules with the calculated dose were prepared by a compounding pharmacy using licensed trilostane. Each owner of a dog enrolled in the study agreed to bring the dog to the hospital for 2 study days, the second no sooner than 3, and no more than 7 days after the first visit. It also was agreed that no change in trilostane dose or frequency of administration would take place between the first and second study days. Diet and environment were kept as stable as possible by the owner of each dog during this intervening period. Any dog evaluated more than once had a minimum of 60 days between the 2 pairs of assessment days. Repeat assessments were evaluated independently.

### 2.3 Study protocol

Other than not receiving trilostane, the healthy dogs were evaluated in the same manner as the trilostane‐treated PDH dogs. Two different sets of evaluation were completed on all dogs. At each evaluation, a complete physical examination was performed after asking the owner about the dog's general well‐being. Owners of PDH dogs were specifically questioned on the first study morning regarding their dog's response to trilostane, understanding that their primary reason for pursuing treatment was resolution of clinical signs. From that owner‐veterinarian conversation, each veterinarian confirmed with the owner 1 of 3 subjective conclusions: that their dog's clinical signs had resolved (adequate dose, A), that their dog was symptomatic and likely would benefit from additional medication even if some improvement in PU, PD, PP or some combination of these had been noted (underdose, U), or that their dog was ill (decreased or no appetite, vomiting, diarrhea or some combination of these) and possibly overdosed (O). Any dog considered ill by the owner or considered ill after the physical examination was to be removed from the study and treated as necessary.

Dogs were randomly assigned to be first evaluated with either the “a” or “b” protocol. Owners were asked to refrain from feeding their dogs on study days and to bring food and trilostane to the hospital with their dogs 60 minutes before trilostane was to be administered. This time might have allowed some dogs to become acclimated to the hospital while the owner history was obtained. Urine, collected and brought in by the owner on the first of 2 study mornings, was assessed for USG, analytes, and UCCR. On study day “a,” 2 hours after trilostane administration, blood was obtained for CBC, serum biochemistry profile, and baseline SCC. Adrenocorticotropic hormone (Nuvacthen. Afasigma S.P.A. Via Ragazzi, Bolonia. Italy) then was administered (5 μg/kg, IM) and blood collected 1 hour later. On study day “b,” each dog had an IV catheter placed 15 minutes before approximately 1 mL of blood was obtained for SCC 30 minutes (−0.5 hour) before trilostane administration. Samples also were obtained via the catheter immediately before (0 hour) and 1, 2, 2.5, 3, 3.5, 4, 6, 8, and 12 hours after trilostane was given. The 12‐hour blood sample was obtained immediately before trilostane was again given and also could be considered a 0 hour SCC.

### 2.4 Assessments

Owner opinion (A, U) was the standard against which the studied variables were assessed. Those variables included results of USG, UCCR, ACTHst, pretrilostane basal SCCs, and post‐trilostane serial SCCs. Often‐used criteria for 2 tests also were compared with owner opinion: USG ≤ 1.019 = U and USG ≥ 1.020 = G; post‐ACTHst SCC ≤ 1 μg/dL = too low; 1.0 to 5.5 μg/dL = A; and >5.5 μg/dL = U.

### 2.5 Hormone assays

Serum and urine cortisol concentrations were measured using of a commercial cortisol assay (Immulite 2000, Siemens Healthcare Diagnostics, Cornellà del Llobregat, Barcelona, Spain) that has been validated for use in dogs. The sensitivity of this assay was 0.5 μg/dL (13.8 nM/L).

### 2.6 Statistical analysis

Statistical analyses were performed using commercially available software (SPSS 25.0 for Windows, SPSS, Inc, Chicago, Illinois). Normal distribution was assessed using the Shapiro‐Wilk test. Because most of the data were not normally distributed, nonparametric tests were performed. When mean values were compared, the Mann‐Wilcoxon test was performed (results expressed as mean ± SD [±SD]). Spearman's rank correlation test was used to determine correlation between continuous variables. Differences were considered significant at *P* < .05.

## 3 RESULTS

### 3.1 Dogs

Three healthy and 22 PDH dogs met their respective inclusion criteria. The healthy dogs (5, 6, and 8 years of age) included 2 females (1 intact in anestrus and 1 spayed) and 1 neutered male. Their body weights were 15 kg, 17 kg, and 19 kg, respectively. The PDH dogs were 8 to 15 years of age (median, 11 years) and included 9 females (5 intact) and 13 males (7 neutered) with body weights of 2.5 to 24.5 kg (median, 11.5 kg). Twenty‐nine evaluations were completed: 3 on healthy dogs and 26 in 21 of the PDH dogs; 1 dog was ill and not evaluated; 3 dogs were evaluated more than once. Two dogs were evaluated 3 times (1 was categorized as A the 3 times and the other A, U and A) and 1 dog was evaluated twice (U first and A second).

Except for the ill dog, the CBC, serum biochemistry, and urinalysis results from each dog were within reference limits or had alterations expected in dogs with HAC.

### 3.2 Classification and trilostane doses

Based on owner observations, the 1 ill dog was removed from the study but classified as a possible overdose, 7 were placed in the A group, and 19 in the U group. The suspected overdosed dog had a brief history of poor appetite and vomiting. At that time, serum electrolyte concentrations were within reference limits, ACTHst results were not consistent with hypocortisolism (pre‐SCC, 1.6 μg/dL; post‐SCC, 5.7 μg/dL), and an alternative diagnosis (gastritis) was made. Because it was the only ill dog and because it was not overdosed, those test results were excluded.

**FIGURE 1:**
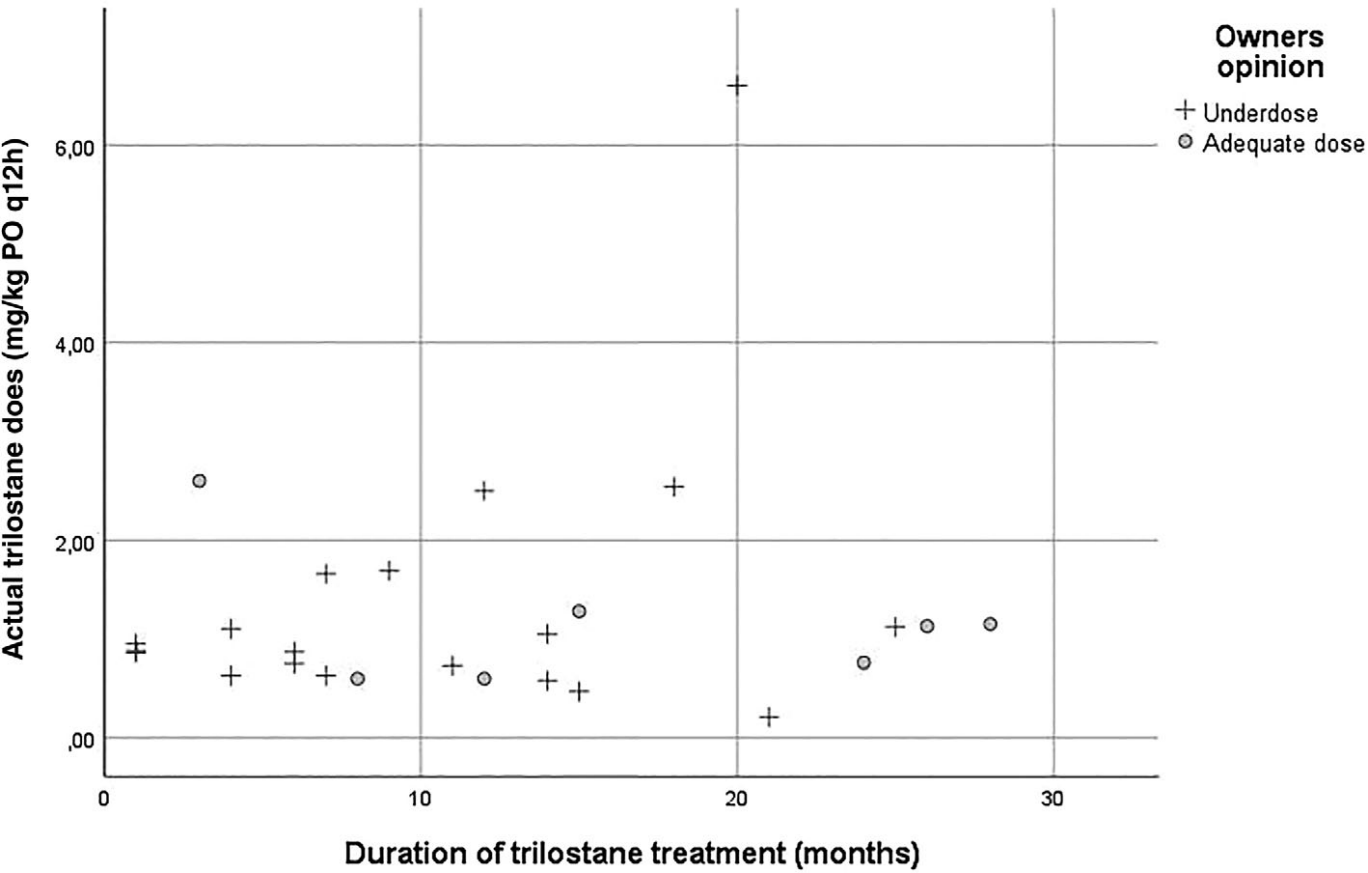
Trilostane dose versus duration of trilostane treatment in dogs considered to be underdosed (U) and to be adequately dosed (A)

The mean duration of trilostane treatment for 21 PDH dogs studied was 8.6 months (range, 1‐25) and dosages at the time of the studies ranged from 0.2 to 6.6 mg/kg PO q12h (mean, 0.91 mg/kg PO q12h). Dogs in the A group had been given trilostane for 3 to 28 months (mean, 13.3 months) and dogs in the U group 1 to 28 months (mean, 7.2 months). Duration of treatment was not significantly different between A and U groups (*P* > .05). Trilostane dosage did not differ between A group (range, 0.6‐2.6 mg/kg; median, 1.13 mg/kg) and U group (range, 0.2‐6.6 mg/kg; median, 0.88 mg/kg; *P* > .05). As seen in Figure 1, 9 of the 11 dogs treated <10 months were in the U group whereas 3 of the 4 dogs treated >24 months were in the A group. Also, as seen in Figures 1 and 4 dogs were receiving >2 mg/kg of trilostane PO q12h, 3 of which were in the U group.

### 3.3 ACTHst, USG, and UCCR

**FIGURE 2:**
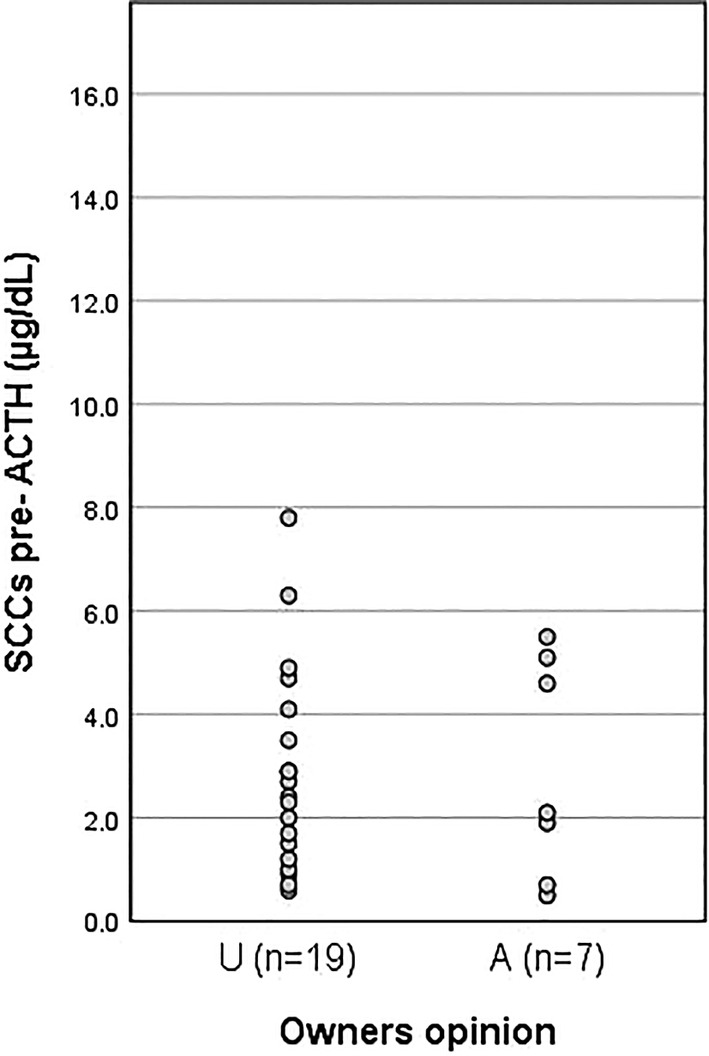
Results of the pre‐ACTH cortisol concentrations for dogs included in the study. First column represents cortisol values for dogs in the underdosed (U) group and the second column the values for the dogs in the adequately dosed (A) group

**FIGURE 3:**
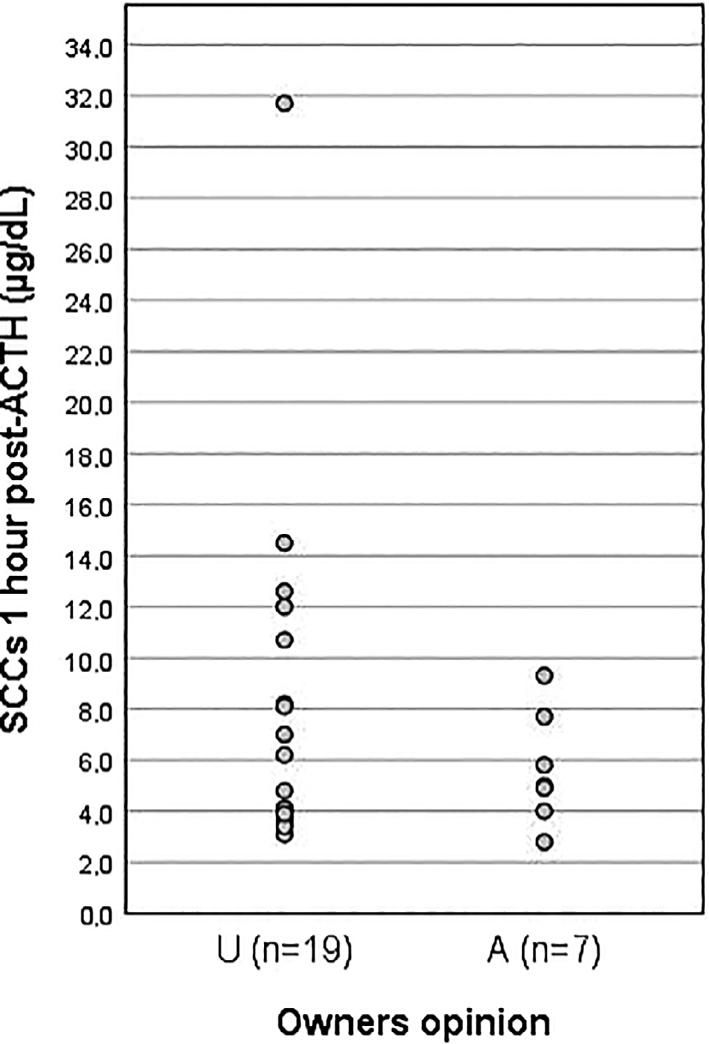
Results of the post‐ACTH cortisol concentrations for dogs included in the study. First column represents cortisol values for dogs in the underdosed (U) group and the second column the values for the dogs in the adequately dosed (A) group

No significant differences (*P* > .05) were found in ACTHst SCC results comparing dogs in the A group with those in the U group (Figures 2 and 3). The mean (±SD) pre‐ACTHst SCC was 2.91 ± 2.11 μg/dL (range, 0.5‐5.5 μg/dL; 2/7 results <1.0 μg/dL) in A dogs and 2.85 ± 1.97 μg/dL (range, 0.6‐7.8 μg/dL; 3/19 results <1.0 μg/dL) in U dogs.

The mean (±SD) post‐ACTHst SCCs were 5.64 ± 2.21 μg/dL (range, 2.8‐9.3 μg/dL) in A dogs and 7.86 ± 6.78 μg/dL (range, 3.1‐31.7 μg/dL) in U dogs; 15 of the 19 U dogs had post‐ACTHst SCCs <9.3 μg/dL, the highest result in the A group (Figure 3). The post‐ACTHst SCC results in 5 of 7 (71%) A dogs and in 9 of 19 (48%) U dogs were between 1.0 and 5.5 μg/dL. The results of the pre‐ACTHst SCC on “day a” and the SCC at 2 hours post‐trilostane on “day b” (the same post‐trilostane timing) for all PDH dogs were not significantly different (*P* > .05).

**FIGURE 4:**
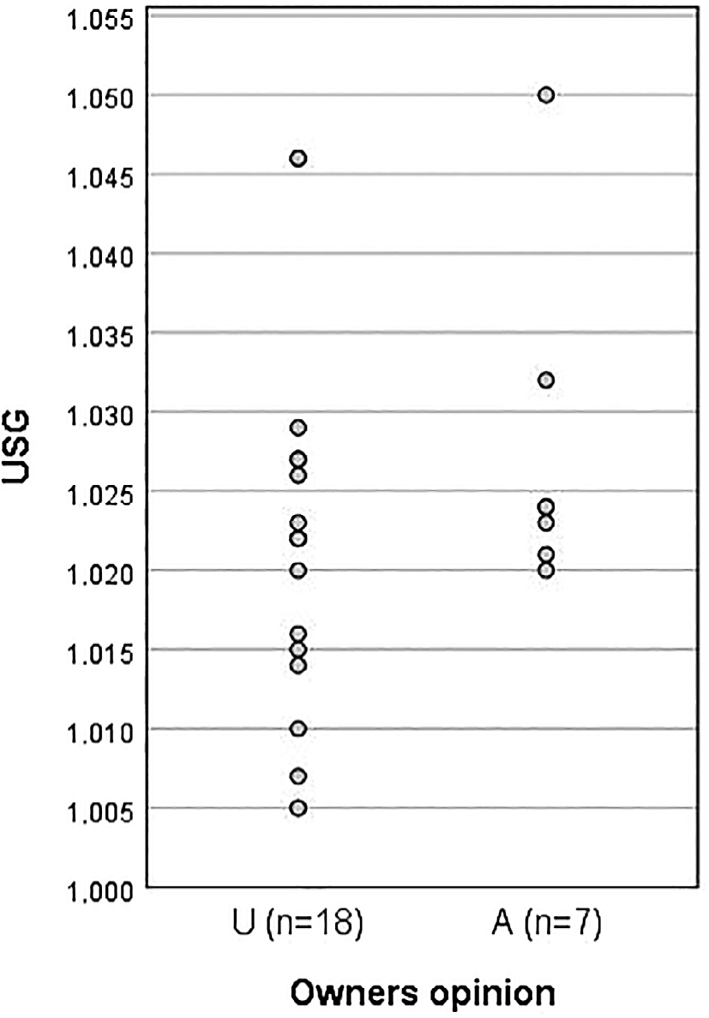
Results of USG for dogs included in the study. First column represents cortisol values for dogs in the underdosed (U) group and the second column the values for the dogs in the adequately dosed (A) group. USG, urine‐specific gravity

**FIGURE 5:**
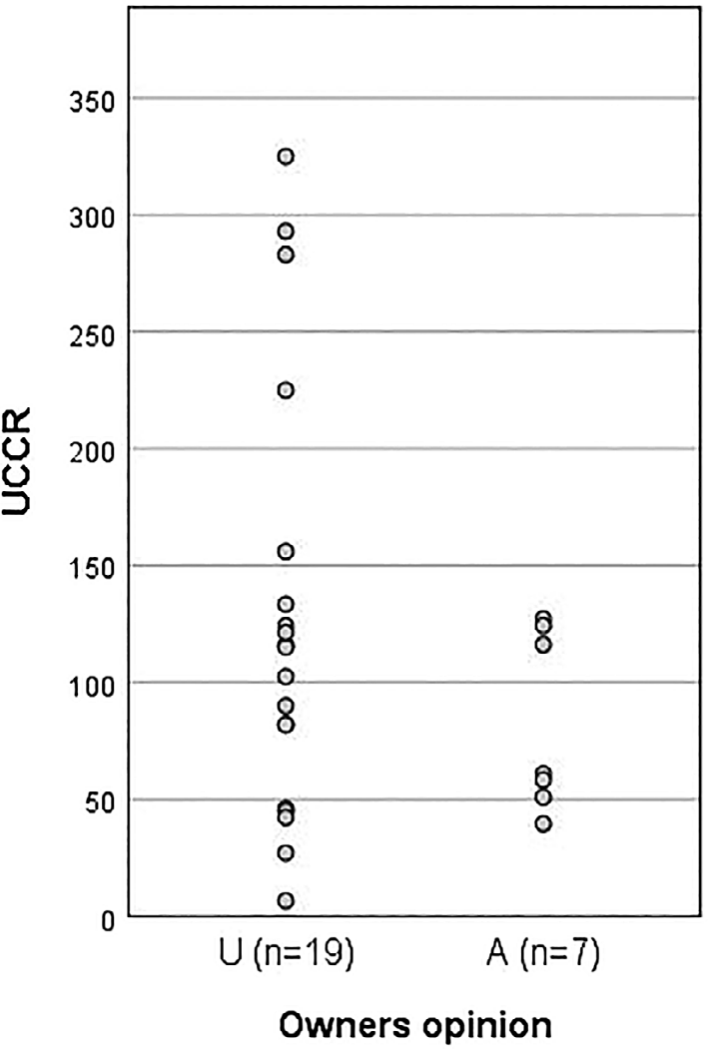
Results of UCCR for dogs included in the study. First column represents cortisol values for dogs in the underdosed (U) group and the second column the values for the dogs in the adequately dosed (A) group. UCCR, urine cortisol: creatinine ratio

No significant difference (*P* > .05) in USG was found comparing the A group results (mean, 1.027 ± 0.01; range, 1.020‐1.050) with the U group results (1.021 ± 0.01; range, 1.005‐1046; Figure 4). The USG in all 7 A and 11 of 19 U dogs was ≥1.020. No significant difference (*P >* .05) was found in UCCR results comparing the A group (82.49 ± 38.23; range, 40‐127) with the U group (127.1 ± 92.1; range, 7‐325; Figure 5). The UCCR was ≤130 in 7/7 A dogs and in 13/19 U dogs.

Five of 7 dogs classified by their owners as A (71%) had ACTHst results of 1.0 to 5.5 μg/dL and USG ≥ 1.020, meeting all 3 commonly used criteria for an adequate dose (owner opinion, ACTHst, USG). Four of 19 dogs classified by their owners as U (21%) had ACTHst results ≥5.5 μg/dL and USG ≤ 1.020, meeting all 3 commonly used criteria for being underdosed.

### 3.4 Serial SCCs from the 3 healthy and 26 PDH dogs

**FIGURE 6:**
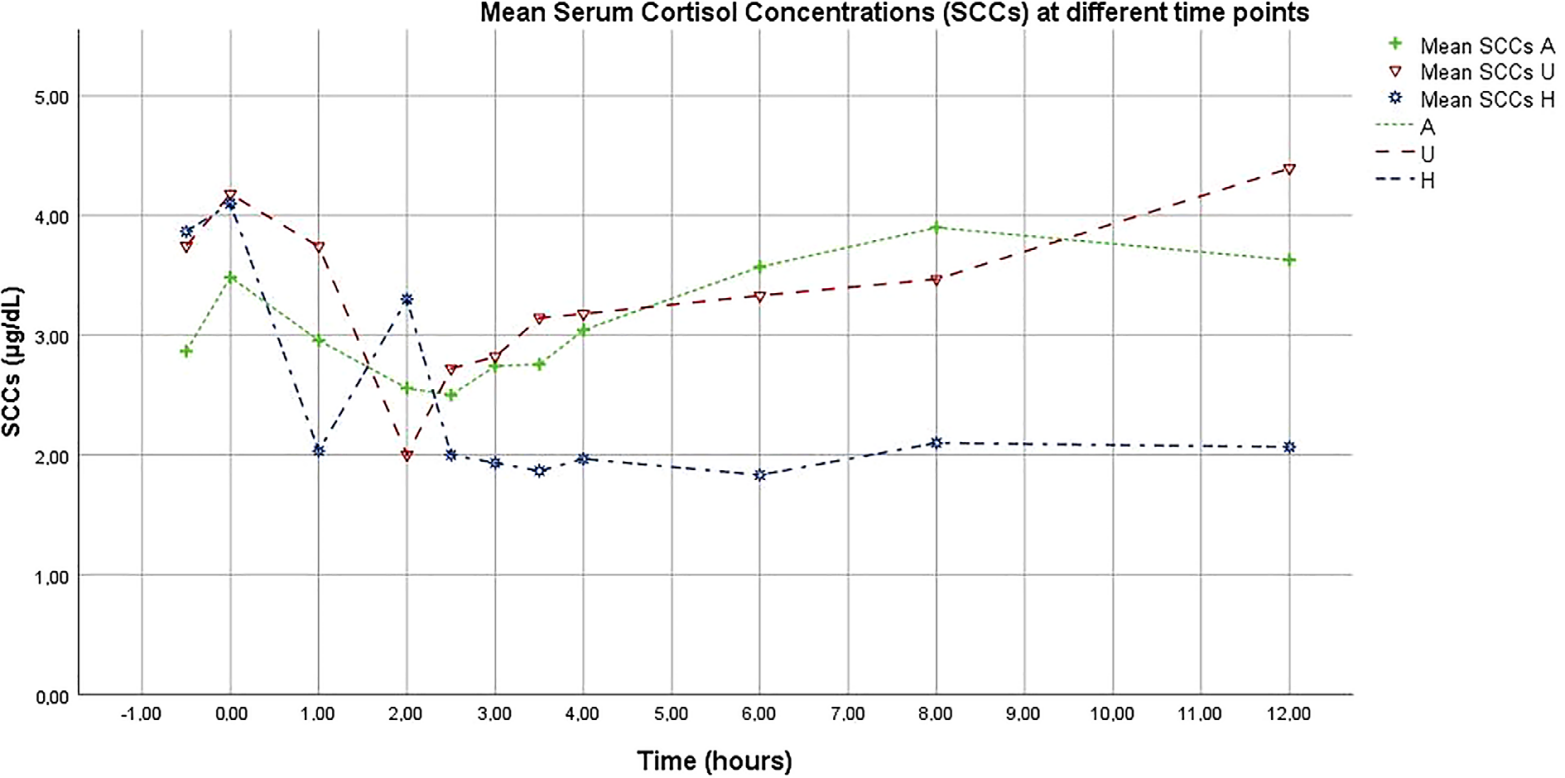
This graphs represents the cortisol curve performed on “day b.” SCC throughout the day for dogs in the adequately dose (A), underdosed (U) group, and healthy (H). The healthy dogs did not receive trilostane. SCC, serum cortisol concentration

**FIGURE 7:**
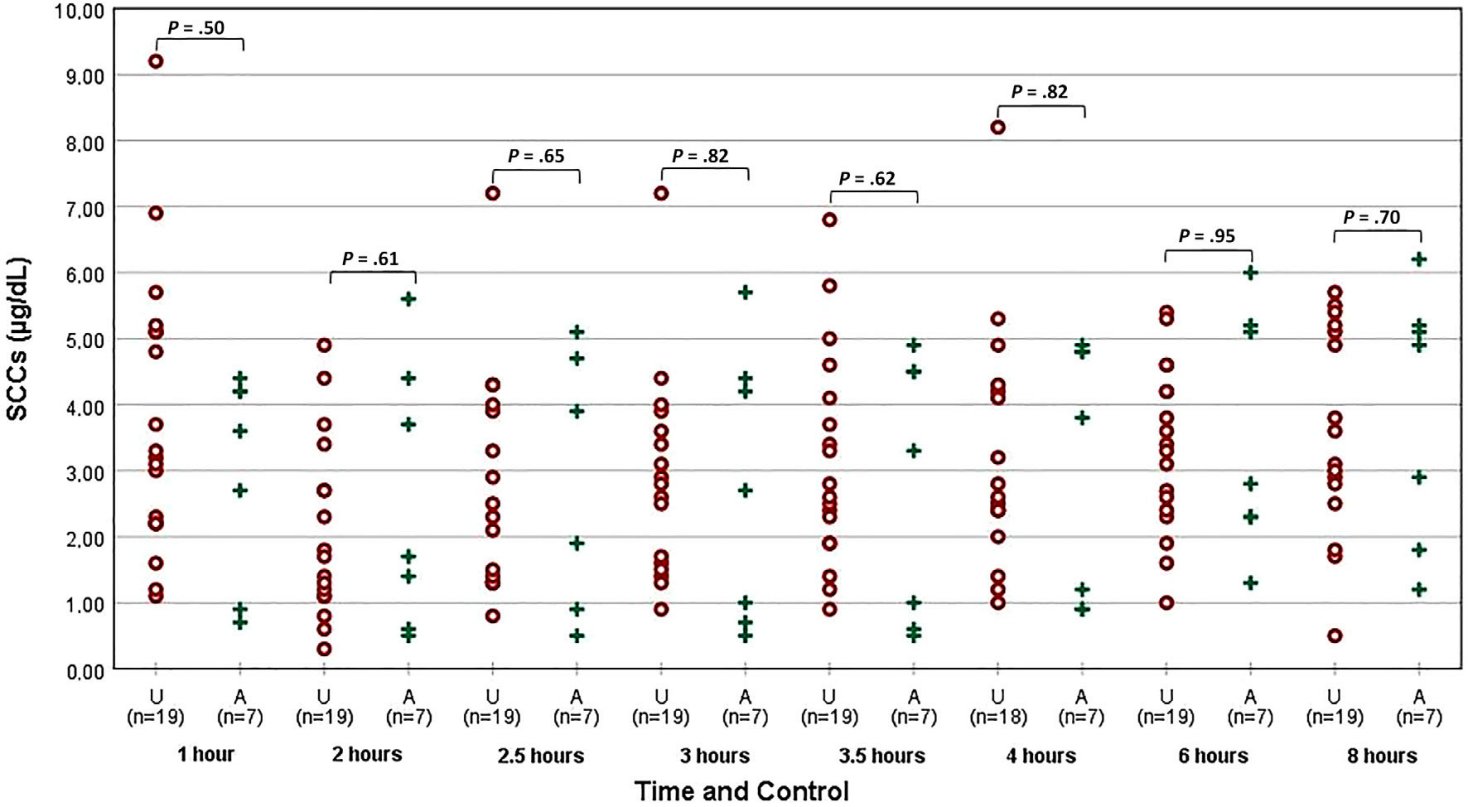
Results of the SCC throughout the day of each dog divided based on owners opinion in adequately dosed (A) and underdosed (U) groups. SCC, serum cortisol concentration

**FIGURE 8:**
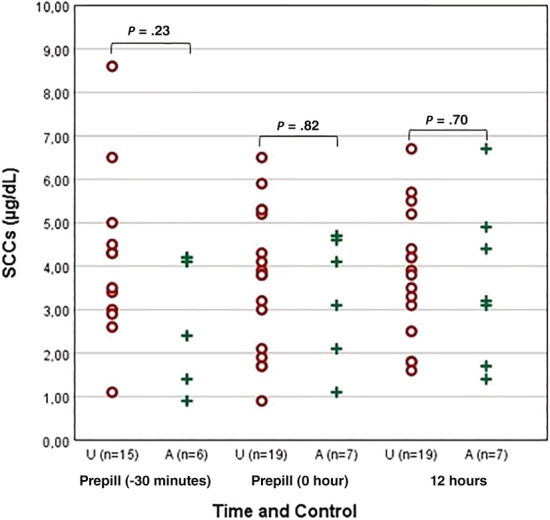
Results of the pretrilostane SCC of each dog divided based on owners opinion in adequately dosed (A) and underdosed (U) groups. SCC, serum cortisol concentration

Mean SCCs at each time point for the 3 healthy dogs and the 26 PDH dog visits are presented in Table 1 and Figure 6. Mean SCCs at 2 and 2.5 hours post‐trilostane administration in the PDH dogs were not significantly different. Their mean SCCs at 2.5 and 3 hours also were not significantly different. In the PDH dogs, the SCC at 2 hours was significantly lower (*P* < .05) than at 3 hours. The SCCs at 2, 2.5, and 3 hours were significantly lower (*P* < .05) than SCCs at −30, 0, 1, 3.5, 4, 6, 8, and 12 hours (Table 1; Figures 6, 7, and 8).

**TABLE 1:** Results of the serial SCCs (µg/dL) throughout the day

	All dogs	Adequate dose	Underdose	Healthy
−0.5 h(prepill)	3.49 ± 1.90 (0.90‐8.60)^c^	2.87 ± 1.50 (0.90‐4.20)	3.75 ± 2.04 (1.10‐8.60)	3.87 ± 1.62 (2.0‐4.80)
0 h (prepill)	3.99 ± 2.36 (0.90‐13.00)^c^	3.49 ± 1.43 (1.10‐4.70)	4.18 ± 2.64 (0.90‐13.0)	4.10 ± 2.12 (2.50‐6.50)
1 h	3.53 ± 1.97 (0.70‐9.20)	2.96 ± 1.58 (0.70‐4.40)	3.74 ± 2.10 (1.10‐9.20)	2.03 ± 0.91 (1.20‐3.0)
2 h	2.15 ± 1.50 (0.30‐5.60)^a^ ^,^ ^b^	2.56 ± 2.00 (0.50‐5.60)	2.00 ± 1.30 (0.30‐4.90)	3.30 ± 1.83 (1.30‐4.90)
2.5 h	2.66 ± 1.65 (0.50‐7.20)^b^	2.50 ± 2.02 (0.50‐5.10)	2.72 ± 1.56 (0.80‐7.20)	2.00 ± 0.85 (1.10‐2.80)
3 h	2.80 ± 1.62 (0.50‐7.20)^a^ ^,^ ^b^	2.74 ± 2.08 (0.50‐5.70)	2.82 ± 1.50 (0.90‐7.20)	1.93 ± 0.71 (1.30‐2.70)
3.5 h	3.03 ± 1.69 (0.50‐6.80)	2.76 ± 1.99 (0.50‐4.90)	3.14 ± 1.62 (0.90‐6.80)	1.87 ± 0.31 (1.60‐2.20)
4 h	3.14 ± 1.77 (0.90‐8.20)	3.04 ± 1.95 (0.90‐4.90)	3.18 ± 1.76 (1.0‐8.20)	1.97 ± 0.59 (1.30‐2.40)
6 h	3.39 ± 1.36 (1.0‐6.0)	3.57 ± 1.82 (1.30‐6.0)	3.33 ± 1.22 (1.0‐5.40)	1.83 ± 0.51 (1.40‐2.40)
8 h	3.58 ± 1.59 (0.50‐6.20)	3.90 ± 1.92 (1.20‐6.20)	3.47 ± 1.51 (0.50‐5.70)	2.10 ± 1.22 (1.30‐3.50)
12 h (prepill)	4.18 ± 3.20 (1.40‐18.0)	3.63 ± 1.86 (1.40‐6.70)	4.39 ± 3.60 (1.60‐18.0)	2.07 ± 0.47 (1.70‐2.60)

*Note:* The results are expressed as the mean ± SD (range).

Abbreviation: SCCs, serum cortisol concentrations.

^a^ Cortisol at 2 hours significantly lower than cortisol at 3 hours (*P* < .05).

^b^ Cortisol at 2, 2.5, and 3 hours significantly lower than −0.5, 0, 1,3.5, 4, 6, 8, and 12 hours (*P* < .05).

^c^ Cortisol at 0.5 hour significantly lower than cortisol at 0 hour (*P* = .01).

The mean prepill SCC at −0.5 hour for all PDH dogs was significantly lower than at 0 hour (*P* = .01) and no significant difference (*P* > .05) was found between their prepill SCC at 0 hour and their prepill SCC at 12 hours. The pretrilostane SCC means of A dogs and U dogs were not significantly different (*P* > .05; Figure 8). There was overlap in results of the SCC from both groups (Figures 6, 7, and 8). No results from a single time or times consistently discriminated between groups but 17/18 pretrilostane A results were <5.0 μg/dL, a sensitive but not specific finding. Of the 12 pretrilostane SCCs >5 μg/dL, 11 were from U dogs, somewhat specific but not sensitive. No correlation was found between prepill SCCs (−0.5, 0, and 12 hours) and nadir SCC at 2 hours (*R* = 0.10, *P* = .6; *R* = 0.45, *P* = .9; *R* = −0.7, *P* = .7).

As seen in Table 1 and Figures 6 and 7, SCCs decreased approximately 2 hours post‐trilostane administration in all treated dogs, followed by increasing SCCs thereafter (Figure 6).

## 4 DISCUSSION

Our aim was to assess the reliability of several commonly used objective monitoring variables for discriminating trilostane‐treated dogs with PDH receiving an adequate dose that effectively ameliorated clinical signs from underdosed dogs with clinical signs of cortisol excess. In addition, the dogs' SCCs were measured serially beginning 30 minutes before trilostane administration through 12 hours after to identify an ideal time for obtaining results most likely to effectively discriminate A from U dogs. No variable assessed in our study consistently correlated with owner opinion and none provided consistent results that discriminated dogs receiving an adequate trilostane dose from dogs that might benefit from an increase in dose or frequency of administration or both.

Our working hypothesis was that USG results would discriminate dogs receiving an adequate dose (>1.020) from underdosed dogs (<1.020). It was assumed that well‐controlled dogs would no longer be PU/PD, which would be reflected by concentrated urine whereas underdosed dogs would remain polyuric and have dilute urine. However, not only did all A dogs have USG >1.020 as anticipated, but 11 of 19 U dogs also had USG >1.020. Thus, USG > 1.020 was a sensitive but not specific indicator of dogs receiving an A dose, rejecting our hypothesis. Conceivably, any dog receiving trilostane could have a second condition causing PU/PD, but dogs with common comorbidities associated with PU/PD were not enrolled in our study. In agreement with other studies, UCCR results did not consistently discriminate A from U dogs.^25^ The only UCCR results >130 (7 results), however, were from U dogs.

Results of ACTHsts in our study did not correlate with clinical signs and did not consistently identify dogs that might benefit from dose adjustment, in agreement with several previous studies.^13^, ^14^, ^16^, ^18^, ^19^, ^20^ These conclusions could have been different had a different time been used for beginning the test. There appears to be only 1 potential scenario in which ACTHst results may be helpful to veterinary clinicians treating dogs with trilostane (ie, to determine if a sick dog is overdosed). In our study, none of the 22 PDH dogs in 27 visits were overdosed. Thus, no observation regarding testing in overdosed dogs is possible except that no A or U dog had a post‐ACTHst SCC <1 μg/dL (none were <2.8 μg/dL). An analysis of trilostane overdosed dogs will be important in future studies. Absence of overdosed dogs in our study may support the concept of initially using conservative doses of trilostane and then titrating dose to the needs of the dog.^14^ The number of U dogs (19) versus A dogs (7), however, suggests that such low starting doses may extend the time needed to resolve clinical signs. As seen in Figure 1, of the 11 studies on dogs treated <10 months, 9 were described by owners as U, whereas 3 of 5 studies on dogs treated >20 months were described as A. Low initial doses and the additional time required to achieve satisfactory control could lead to owner disappointment, frustration, and dissatisfaction. The issue of starting dosage remains to be better understood.

Some authors have evaluated pretrilostane‐administration SCCs to assess HAC control and need for dose adjustment.^21^, ^33^, ^34^ This approach suggests that SCCs after trilostane effects have waned will provide objective information regarding the dose and frequency needs of the dog. Results of 1 study suggested that pre‐trilostane and 3 hour post‐trilostane SCCs potentially were better monitoring variables than ACTHst results,^21^ but many studies have suggested ACTHst results to be of questionable monitoring value. Furthermore, there was no clear indication of when the pretrilostane SCCs were measured relative to timing of the next dose or relative to the timing of arrival at the hospital. In a subsequent study, pretrilostane SCCs were found to be more consistent and reliable than 3 hour post‐trilostane SCCs.^33^ Based in part on these observations, measurement of the pretrilostane SCC has been advocated as a reliable and informative variable for monitoring trilostane‐treated dogs.^21^

Our results do not concur with these observations and recommendations. Reviewing the 26 serial cortisol studies, there was no time point in which SCCs discriminated A from U dogs, including the −0.5, 0, and 12 hours SCCs. The SCCs measured before and after trilostane administration overlapped (Figures 6, 7, and 8). Similarly, another study found only moderate agreement when comparing 2 pretrilostane SCCs taken 1 hour apart, and 30% of those dogs had paired results, each of which would have led to completely different treatment decisions.^34^ Adding to this confusion, the first SCCs obtained from all groups of dogs in our study were significantly lower than those obtained 30 minutes later. The difference in SCC throughout the day was even more striking in healthy dogs for which evening SCC was lower than morning SCC. This difference could be a reflection of cortisol circadian rhythm in healthy dogs or a reflection of dogs becoming accommodated to the hospital environment. Initial nervousness or anxiety upon entering the veterinary hospital in some dogs may have an important role in determining SCC, and decreasing cortisol concentrations throughout the day might indicate calming over time.

The serial SCCs obtained from the 26 visits profile trilostane's effect in blocking cortisol synthesis and secretion. The SCCs results of all 26 visits were significantly lower at 2, 2.5, and 3 hours after administration than at all other time points. Using a q12h schedule, trilostane's effect begins within an hour and peaks approximately 2 to 3 hours after administration. Trilostane's effect appears to have completely dissipated in most dogs within each 12 hour‐period. For most profiles, as seen in Figure 6, trilostane's effect begins to wane by approximately 4 hours and its effect does not commonly persist beyond approximately 6 to 8 hours. The nadir in SCC for most dogs is expected to be approximately 2 hours, but overlap among A and U dog results precludes identifying an ideal time for obtaining SCC. Both the A and U dogs were being given similar doses of trilostane. However, in reviewing Figure 6, the A dog SCC curve begins lower, decreases sooner and more gradually, and dissipates more gradually than the results in the U group. The SCCs in the U dogs remain static longer, then decrease more dramatically after trilostane administration, and increase sooner and more dramatically than what was observed in the A dogs. Perhaps, the need for larger doses in some dogs might have been the result of a more transient effect in that population or the more dramatic and transient decrease in the U dogs' SCC could be consistent with overdose rather than underdose. Furthermore, some U dogs might have benefited from q8h dosing. Results of our study and of previous studies do not identify a variable that is useful in making these decisions.

Our results and those of many previous studies support the need for individualizing treatment, regardless of initial dose, to the needs of the dog as noted by the owner. It seems logical that trilostane dose requirements would vary somewhat from dog to dog. The trilostane used in our study was from a single source, eliminating 1 explanation for different responses to similar doses. Because drug source was not an issue, these results suggest that it may be of value to assess the diets or the gastrointestinal health of A and U dogs. A problem in 1 of these areas could enhance or interfere with drug absorption or action. In the dogs studied here, no other medications were being given (another possible reason for different drug activity profiles).

Our assumption was that owners would best know their dogs and whether their dogs, clinical signs of PDH had resolved completely, partially, or not at all. Use of owner opinion as the variable against which objective testing was assessed was based on the realization that most dogs with HAC develop intolerable clinical signs. Those signs are the most common reason for bringing a dog with PDH to a veterinarian and the reason for treatment. This approach does not ignore the many physiological benefits of treatment, rather it is appreciated that resolution of clinical signs usually correlates with the physiological benefits. Thus, owner opinion, although not always perfect, remains a key variable to understand and utilize when deciding whether a dose change is indicated. Finally, USG may have value as a sensitive (but not specific), simple, quick, and inexpensive indicator of appropriate dose and frequency.

Limitations of our study include the relatively small number of PDH dogs in each owner opinion category. Perhaps control of diet, feeding times, or other factors in the home environment would be beneficial. A satisfactory owner questionnaire might have provided important information, but we believe no questionnaire would change overall owner appraisal of the dog's response to trilostane. Retrospectively, an additional time point SCC assessment at 11.5 hours of the serial cortisol concentration study also would have provided additional data regarding sampling “30 minutes before” trilostane administration. As discussed, a study of overdosed dogs will be important as would a study of dogs with adrenal‐dependent hyperadrenocorticism.

Many of the tests most commonly used in monitoring dogs being treated with trilostane for PDH were included in our study. Furthermore, serial SCCs also were obtained beginning before and extending 12 hours after trilostane administration. No variable consistently discriminated dogs receiving an adequate dose from dogs that continued to exhibit clinical signs. As discussed, these results support the importance of knowing the owner's and veterinarian's goals for treatment, and these should be aligned before beginning treatment. Based on results of our study and many others, veterinarians treating dogs with trilostane for PDH are encouraged to develop an effective team approach. Valuable members of the team include the owner in addition to the veterinarian. Adequate time must be available for both team members when reevaluating these dogs so as to fully understand owner observations.

## CONFLICT OF INTEREST DECLARATION

Authors declare no conflict of interest.

## OFF‐LABEL ANTIMICROBIAL DECLARATION

Authors declare no off‐label use of antimicrobials.

## INSTITUTIONAL ANIMAL CARE AND USE COMMITTEE (IACUC) OR OTHER APPROVAL DECLARATION

Approved by the hospital board of the Veterinary Teaching Hospital Complutense, Las Palmas de Gran Canaria and from the Hospital Auna Especialidades Veterinarias.

## HUMAN ETHICS APPROVAL DECLARATION

Authors declare human ethics approval was not needed for this study.
